# Sensitive and reliable detection of Kit point mutation Asp 816 to Val in pathological material

**DOI:** 10.1186/1746-1596-2-37

**Published:** 2007-09-27

**Authors:** Christian Kähler, Sabine Didlaukat, Alfred C Feller, Hartmut Merz

**Affiliations:** 1 Institute of Pathology, University of Luebeck, Ratzeburger Allee 160, 23538 Luebeck, Germany

## Abstract

**Background:**

Human mastocytosis is a heterogenous disorder which is linked to a gain-of-function mutation in the kinase domain of the receptor tyrosine kinase Kit. This D816V mutation leads to constitutive activation and phosphorylation of Kit with proliferative disorders of mast cells in the peripheral blood, skin, and spleen. Most PCR applications used so far are labour-intensive and are not adopted to daily routine in pathological laboratories. The method has to be robust and working on such different materials like archival formalin-fixed, paraffin-embedded tissue (FFPE) and blood samples. Such a method is introduced in this publication.

**Methods:**

The Kit point mutation Asp 816 to Val is heterozygous which means a problem in detection by PCR because the wild-type allele is also amplified and the number of cells which bear the point mutation is in most of the cases low. Most PCR protocols use probes to block the wild-type allele during amplification with more or less satisfying result. This is why point-mutated forward primers were designed and tested for efficiency in amplification of the mutated allele.

**Results:**

One primer combination (A) fits the most for the introduced PCR assay. It was able just to amplify the mutated allele with high specificity from different patient's materials (FFPE or blood) of varying quality and quantity. Moreover, the sensitivity for this assay was convincing because 10 ng of DNA which bears the point mutation could be detected in a total volume of 200 ng of DNA.

**Conclusion:**

The PCR assay is able to deal with different materials (blood and FFPE) this means quality and quantity of DNA and can be used for high-througput screening because of its robustness. Moreover, the method is easy-to-use, not labour-intensive, and easy to realise in a standard laboratory.

## Background

1,000 to 8,000 incidences of human mastocytosis are reported every year in the US [1]. Human mastocytosis is characterised by accumulation of mast cells in different organs. It is a heterogenous group of disorders which can be divided into the categories cutaneous mastocytosis (CM) and systemic mastocytosis (SM) which is commonly seen in adults by histological lesions in the bone marrow and other non-cutaneous organs [2,3]. SM can be further divided into the categories indolent systemic mastocytosis (ISM), SM with an associated clonal hematologic non-mast cell lineage disease (AHNMD), aggressive sytemic mastocytosis (ASM), and mast cell leukemia (MCL). ISM is the most common form which involves skin, bone marrow, and GI tract with good prognosis for the patient. First relations between mastocytosis and activating mutations in the receptor tyrosine kinase Kit came from the human mast cell line HMC-1 [4]. A gain-of-function mutation in the kinase domain of Kit (D816V) leads to constitutive tyrosine kinase activation and phosphorylation of Kit. The consequence is a ligand-independent cell proliferation. It has been shown that this point mutation causes mastocytosis of peripheral blood lymphocytes and skin and spleen mast cells [5,6].

There is a strong need for a reliable and sensitive method for detection of the Kit point mutation Asp 816 to Val which matches the needs for high-throughput screening of blood and FFPE samples. In this publication we present an easy-to-use, unexpensive, and reliable method for detection of this mutation.

## Methods

### Samples

The human mast cell line HMC-1 is heterozygous for the D816V point mutation and was obtained directly from P. Valent (University of Vienna). HMC-1 was used as a positive control in all experiments and DNA from tonsil as a negative control. The presence of Kit mutations was investigated in archival formalin-fixed, paraffin-embedded tissue (FFPE) from five patients as well as blood samples from five patients also. All experiments were carried out in accordance to the Helsinki Declaration.

### DNA Extraction

For extraction of total genomic DNA, 20-μm-thick sections were cut from each paraffin block and DNA was extracted by standard methods. In brief, dewaxing was done by xylene and it was followed by overnight proteinase K digestion. For genomic DNA preparation the QIAamp DNA Mini Kit (Cat. No. 51306) was used as well as for all other DNA preparations (e.g. cell lines and patient's blood samples). DNA concentration was obtained by a spectrophotometer (ND-1000, NanoDrop).

### Primers and PCR conditions

Following primer combinations have been used for PCR in combination with the Pure Taq Ready-To-Go PCR beads system of GE Healthcare (Cat. No. 27-9559-01). Beads were supplemented with 10 pmol of each primer, DNA and HPLC grade H_2_O to a final volume of 25 μl.

Primer combination A (Annealing Temperature 57°C)

MastoMutF1: 5'-TGTGATTTTGGTCTAGCCAGAGTG-3'

MastoMutR1: 5'-TGTTCAGCATACCATGCAAA-3'

Primer combination B (Annealing Temperature 55°C)

MastoMutF2: 5'-TGTGATTTTGGTCTAGCCAGAGTA-3'

MastoMutR1: 5'-TGTTCAGCATACCATGCAAA-3'

Primer combination C (Annealing Temperature 56°C)

MastoMutF3: 5'-TGTGATTTTGGTCTAGCCAGAGTT-3'.

MastoMutR1: 5'-TGTTCAGCATACCATGCAAA-3'

WT sequence KIT: 5'-TGT GAT TTT GGT CTA GCC AGA GAC-3'

D816V KIT: 5'-TGT GAT TTT GGT CTA GCC AGA GTC-3'

Mutated forward primers have a 3'-terminal mismatch – G, A or T instead of C – and a second mismatch one base from the 3'-nucleotide which represents the point mutation A→T.

Each PCR cycle consisted of 30 seconds denaturation at 94°C, 30 seconds annealing at the stated temperatures (A 57°C/B 55°C/C 56°C), and 45 seconds elongation at 72°C and was repeated 36 times. The initial denaturation was done for four minutes at 94°C and final elongation for four minutes at 72°C. A 174 bp long fragment was amplified. Electrophoresis was done on a 2% TAE gel and the PCR products were cloned by TOPO TA Cloning Kit (Invitrogen, Part no. 45-0641) and clones were prepared for sequencing using the GenomeLab Dye Terminator Cycle Sequencing Kit (Beckman&Coulter, P/N 608120). Samples were run on the CEQ8800 sequenzer (Beckman&Coulter).

Defining sensitivity of PCR-assay (Table 1)

**Table 1:** 

1. 200 ng gen. DNA HMC-1 (ca. 28571 cells)	0 ng gen. DNA tonsil (ca. 0 cells)	no dilution
2. 100 ng gen. DNA HMC-1 (ca. 14285 cells)	100 ng gen. DNA tonsil (ca. 14285 cells)	1:2
3. 50 ng gen. DNA HMC-1 (ca. 7142 cells)	150 ng gen. DNA tonsil (ca. 21428 cells)	1:4
4. 10 ng gen. DNA HMC-1 (ca. 1428 cells)	190 ng gen. DNA tonsil (ca. 27142 cells)	1:20
5. 1 ng gen. DNA HMC-1 (ca. 142 cells)	199 ng gen. DNA tonsil (ca. 28428 cells)	1:200
6. 0,1 ng gen. DNA HMC-1 (ca. 14 cells)	199,9 ng gen. DNA tonsil (ca. 28557 cells)	1:2000
7. 0 ng gen. DNA HMC-1	200 ng DNA tonsil (ca. 28571 cells)	no dilution

200 ng of genomic DNA was the final concentration in every sensitivity testing.

## Results

**Figure 1:**
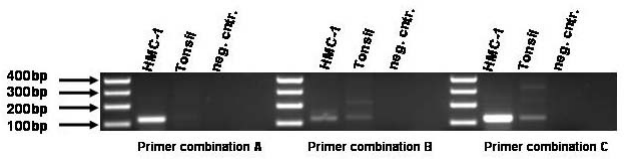
**Testing of point mutated primers**. 200 ng of genomic HMC1 DNA was used as a PCR template for testing the primer combinations. Primer combination A fits the most.

**Figure 2:**
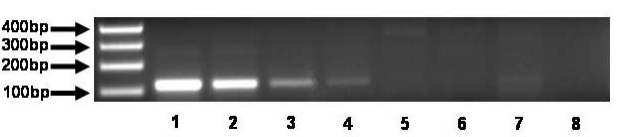
**Defining sensitivity of PCR assay**. Total volume of DNA per PCR 200 ng. Lane 1: 200 ng HMC-1 DNA, Lane 2: 100 ng HMC-1 DNA + 100 ng tonsil DNA, Lane 3: 50 ng HMC-1 DNA + 150 ng tonsil DNA, Lane 4: 10 ng HMC-1 DNA + 190 ng tonsil DNA, Lane 5: 1 ng HMC-1 DNA + 199 ng tonsil DNA, Lane 6: 0,1 ng HMC-1 DNA + 199,9 ng tonsil DNA, Lane 7: 200 ng tonsil DNA, Lane 8: control.

**Figure 3:**
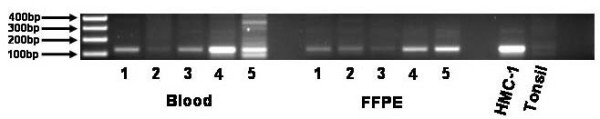
**Detection of point mutation Asp 816 to Val in blood or FFPE samples**. Blood FFPE. Lane 1: 35 ng template 25 ng template. Lane 2: 190 ng template 20 ng template. Lane 3: 189 ng template 224 ng template. Lane 4: 205 ng template 186 ng template. Lane 5: 296 ng template 125 ng template. HMC-1, tonsil (200 ng).

In an initial experiment the most suitable primer combination was determined according to Kwok et al. [7] by testing the three combinations on HMC-1 DNA and tonsil DNA as a wild-type control. Primer combination A fulfilled the criteria of specificity in amplifying only the point mutated allele of HMC-1 (see Fig. 1). B and C showed amplification of the WT control so that they are not useful for this assay. The next question which was addressed was the sensitivity of this PCR based assay. For this reason a dilution series with HMC-1 DNA and tonsil DNA had been done whereas the total DNA concentration of every PCR was 200 ng. The minimum concentration of HMC-1 DNA which was still detectable was 10 ng in a total volume of 200 ng of DNA (see Fig. 2). This means a 1:20 dilution. For further testing of primer combination A on patient's material five blood samples and five FFPE samples were used as a template for PCR. The clinical parameters of all these patients clearly indicate mastocytosis and the question was if the Kit point mutation Asp 816 to Val is the reason for this disease pattern. Due to the heterogeneity of patient's samples the DNA in every PCR was variable. At the end the introduced assay with primer combination (A) is able to detect the point mutation in all samples of formalin-fixed, paraffin-embedded tissue (FFPE) and blood (see Fig. 3).

## Discussion

Several methods have been implemented before to detect the point mutation D816V in Kit as a diagnostical proof for mastocytosis. All of them are highly sensitive PCR-based methods because of the typically low number of cells bearing the point mutation. In 1995 Nagata et al. [5] published a PCR for amplification of introns 16 and 17 of Kit on DNA and mRNA level of peripheral blood mononuclear cells. The amplicon includes D816V which introduces an A to T substitution at nt 2468 and a new HinfI restriction site. After digestion of the amplicon and the wt amplicon the two patterns can be compared. The disadvantage of this method is that the transfer from blood samples to FFPE tissues did not work well because of the limited quality of DNA (data not shown). Sotlar et al. [8] published a modified method [9] for mutation detection in FFPE material by the help of PNA directed PCR clamping. The PNA suppressed the amplification of the wild-type target even when the number of tumour cells is very low in the whole cell fraction. The read-out of this assay was done in the LightCycler with additional hybridization probes. Taken all this in account the method needs expensive lab equipment and chemicals. Tan et al. [10] introduced a so-called allele-specific competitive blocker PCR (ACB-PCR) which needs a modified primer and which was very similar to the allele-specific PCR of Corless et al. [11] which also needs a blocking primer. We developed a simple PCR assay based on the publication of Kwok et al. [7] and combined it with a Ready-To-Go PCR bead format (GE Healthcare) which is convenient for high-throughput screening. The idea of the PCR is that single internal mismatches in the oligonucleotides have no significant effect on PCR product yield. But primer:template mismatches at the 3'-terminal base reduced the overall PCR product yield about 100-fold when the mismatch is not involving a T at the 3' end and when it is coupled with any additional mismatch 1, 2, or 3 bases upstream from the 3'-nucleotide. This is why three different forward-primers were designed for PCR. All these primers have a 3'-prime mismatch compared to the wild-type sequence. This means for MastoMutF1 G instead of C, MastoMutF2 A instead of C, and MastoMutF3 T instead of C. At every forward primer an additional mismatch was introduced one base from the 3'-nucleotide: the point mutation in Kit. Only the MastoMutF1 primer showed the expected specificity that only the point mutated allele of HMC-1 was amplified. The T mismatch in MastoMutF3 also amplified wild-type allele from tonsil DNA as well as MastoMutF2. Interestingly, MastoMutF2 had the lowest signal intensity overall. For all further experiments the primer combination A with MastoMutF1 as a forward primer was used. Sensitivity testing of this assay revealed that 10 ng in a total volume of 200 ng of DNA could be detected. This means a 1:20 dilution or one mutated cell in a total of 20 cells. It is known that in human somatic cells the DNA concentration is around 7 pg per single diploid cell in G0 phase [12]. 10 ng HMC-1 DNA are around 1428 cells in 27142 tonsil cells (190 ng). Due to the fact that HMC-1 is only heterozygous for the point mutation the assay decribes a high sensitivity.

## Conclusion

In daily routine the amount of available material as well as the handling (e.g. fixation) varies. FFPE is the most widely used material in pathological institutes with consequences for the nucleic acids. During the process of fixation nucleic acids are cross-linked to proteins which is a problem for isolation and often degradation occurs before the material is completely fixed [13]. This is why the quantity and quality of isolated DNA is not identical and the amount of template for PCR differs. Taken together, the introduced assay is able to handle all the differences in DNA material successfully. In summary, the detection of Kit point mutation Asp 816 to Val can be done reliably, sensitively, time-saving, and cost-efficient by the introduced assay.

## Competing interests

The author(s) declare that they have no competing interests.

## Authors' contributions

CK developed the PCR assay and made substantial contributions to analysis and interpretation of data as well as in writing the manuscript.

SD was involved in acquisition of data and analysis.

ACF revised critically the manuscript for important intellectual content.

HM has been involved in drafting the manuscript and has given final approval of the version to be published.

All authors read and approved the final manuscript.
